# Narratives of Aging and the Care Home in South Africa: Insights from Antjie Krog’s *Blood’s Inner Rhyme* (2025)

**DOI:** 10.1007/s10823-026-09604-w

**Published:** 2026-10-21

**Authors:** Oscar Ortega Montero

**Affiliations:** Wits Institute for Social and Economic Research (WiSER), University of the Witwatersrand, Johannesburg, South Africa

**Keywords:** Aging, Care home, Memory, Medical humanities

## Abstract

Aging provides a chronicle of experiences and an opportunity to elaborate on the meaning of life and its course. In *Blood’s Inner Rhyme* (2025), Krog walks the readers through the realities of aging and sheds light on the emotional realm of it, showcasing the interplay between human relations, solitude, and the cumulative impact of the past in the care home. I examine these issues in the context of dementia, approaching the memories that impacted on Krog’s ability to navigate Serfontein’s characterization in later life. My analysis also includes an overview of their lives as a way of constructing meaning out of the patterns that we individuals perceive with the passage of time. Additionally, I seek to prove that humanities-based inquiries are instrumental in exploring the complexities of aging, identity and personal realization that emerge in the care home, thus reflecting on the whimsical nature of memory. To this end, I utilize the theoretical frameworks of life writing, aging studies and medical humanities.

## Introduction

Antjie Krog’s literary contributions have garnered international praise and have been the recipient of multiple writing industry awards, such as the 1999 Alan Paton award for non-fiction and the 2004 Nielsen Booksellers’ award. Her works have also been translated into several languages, including English, Spanish and Arabic, amongst others. Krog’s *oeuvre* covers different genres ranging from poetry to translation and non-fiction. Nonetheless, Krog is better known for her non-fictional writing, namely *Country of My Skull* (1998) and *A Change of Tongue* (2003). Both document the first steps of a new era in South Africa. In her latest work *Blood’s Inner Rhyme* (2025), a powerful autobiographical novel, Krog reflects on her mother’s life, the Afrikaner writer Dot Serfontein, who struggles with the onset of dementia and lives in a care home.

One of the most compelling aspects of the relationship between Krog and her mother lies in the fact that both live in the aftermath of trauma. Krog’s view promotes the reduction of the socioeconomic disparities that developed during apartheid. Serfontein, in contrast, appears to be committed to keeping alive the ideological legacy of apartheid South Africa. The political landscape is therefore precarious. However, as will be discussed later, one of the insights that arises from the examination of this filial relationship in the context of the care home is the staying power of Serfontein’s views in Krog’s mind. This is a core element in a narrative that unpacks suppressed aspects of Serfontein’s life. Krog speaks at great length the defining elements of Serfontein in later life, blending different forms of life writing, such as past memoirs, oral narratives, diaries, photographs and letters to construct a compelling narrative. The story compels Krog to revisit her upbringing in the Free State province. Krog places emphasis on her mother’s early diagnosis of dementia, making room for Serfontein’s muddling of names, cherished memories, memory lapses or occasional outbreaks of violence that are often associated with the early symptoms of dementia.

Krog’s latest work navigates the intricacies of the passage of time and the central role of memory, examining gains and losses. *Blood’s Inner Rhyme* adds to the growing literature that focuses on the realities of age-related conditions while walking the readers through some of the unpleasant aspects of later life (De Falco, 2010; Falcus & Sako, 2019; Sandberg & Ward, 2023; Twigg, 2000; Zimmerman, 2017, 2020). In this regard, the novel provides valuable insights into the meaning of human existence in the context of the care home, thus contributing to scholarly debates on the complexities surrounding age-related issues.

The exploration of aging in Krog’s narrative enables different age groups of readers to have “close-in contact with far-out lives” (Geertz, 1989, pp. 5–6). In this sense, life writing, perhaps more than any other literary genre, allows Krog to deepen her exploration of losses and gains throughout her life (Birren & Deutchman, 1991; Couser, 2012, 2021). As Krog observes, “[l]ife is full of unused opportunities” (2025, p. 207); and these ‘unused opportunities’ merit further consideration. Life writing thus provides us with the necessary means to re-assess the past, taking in mind that with the benefit of hindsight, we can see our mistakes, write about them with certain authority, and more importantly, make amendments. Krog embarks on an emotional path that sometimes turns into a slippery slope, which she traverses with wit and charm. In this way, the author comes to terms with the realities of her aging mother as well as the change in the nature of their relationship, reversing roles gradually as Serfontein’s dementia progresses.

Krog also examines the various challenges that she and her mother face upon early diagnosis of dementia. Thus, Krog addresses a convulsive emotional landscape that comprises a wide range of feelings including instances of anger, despair, tenderness and sincerity as her emotions fumble forward. It is against this backdrop that Krog offers an insightful perspective into the act of witnessing the gradual deterioration of Serfontein’s health, which operates as a metaphor for the loss of values and beliefs that Serfontein’s apartheid South Africa once embodied.

Central to this metaphor is Serfontein’s resistance to abandoning and selling the farm, which criticism sees a symbol in Afrikaner culture and literature (Coplan, 2000; Devarenne, 2009; Marx Knoetze, 2020). The fact that neither Krog nor her siblings can provide adequate care for Serfontein on the farm, where she is entrenched as a symbol of escapism from the current era, provides an arena for the exploration of guilt. This feeling mostly stems from Krog and her siblings’ detachment from farm life. This responds to a shift in cultural patterns in accordance with Krog’s public anti-apartheid spirit and embrace of blackness in her work *Begging to be black* (2009) (Balcomb, 2025; Pujolràs-Noguer, 2024). The fact that Krog openly criticizes Afrikaner rule in South Africa infuriates the matriarch of the family. Serfontein sees her death would be a turning point in her children’s compliance with Afrikaner cultural and identity markers. Krog’s estrangement from the symbolic value attached to the farm in Afrikaner culture acquires greater significance because, as writers, mother and daughter are purveyors of a culture that in the eyes of criticism developed the so-called Afrikaner farm novel or *plaasroman* (Devarenne, 2009; Warner, 2011).

Moreover, Krog’s rejection of and moving away from the farm is a key inflection point as it is tantamount to pushing to the margins white privilege, colonialism, and segregation. As I will show, this element illustrates that the blood’s inner rhyme has to do with the internal migration, abandoning a rural and conservative culture in favour of an urban life symbolizing modernity in the new era. Furthermore, Serfontein’s frailty precipitates her ideological downfall in the family, and her passing away is portrayed as that of “the last true Afrikaner” (2025, p. 231). This underscores the idea that the erasure of the remains of the old South Africa after 1994 has slowly but surely given rise to another, where political culture is still in the making and under scrutiny in the eyes of the matriarch.

The recognition of the rights over land of the formerly disenfranchised, along with the restoration of dignity, becomes a source of conflict. This shift in the perception of land ownership is fueled by the promising prospects of a better South Africa (McLean, 2009; Hugues, 2014). Krog welcomes with her arms wide open the new reality of South Africa. Serfontein, in turn, is reluctant to it, sticking to the idea of South Africa as a geo fantasy that no longer exists. Serfontein’s mindset is propelled by the sometimes-imaginary world that dementia promotes in her. The land is disputed and South Africa is thus again a place of contested boundaries, as in the old colonial days. Serfontein’s choice takes the form of resistance to sell the farm on the grounds of fear of retaliation because, in the words of Serfontein, “[y]ou know they want to expropriate our farm, hey?” (2025, p. 45). Such a belief instills fear, enabling Krog to explore how deeply-ingrained racial prejudice is in the mindset of her mother, who cannot avoid becoming embroiled in the enforcement of the old regime, as if purposely stuck in time.

In this article, I employ the theoretical frameworks of life writing, literary gerontology, and, to a lesser extent, medical humanities to tackle the challenge of dementia care that is present in Krog’s narrative. Likewise, I also seek to address societal attitudes in relation to health, care and social change in South Africa, showcasing the key role of life narratives when individuals and communities are faced with turning points in their lives. This article also seeks to raise awareness on how stories featuring vulnerable subjects further our knowledge and understanding of later life (Eakin, 1999, 2008, 2020).

## Life Writing in the Context of Aging

Life writing is a form of social report that reflects the concerns and curiosities of individuals, thus delving into the multifaceted realities that constitute a challenge to human agency (Beard, 2016; Beard et al., 2009). These intimate forms of narration operate as bridges between individuals willing to share their cosmovision with the outer world, showcasing both societal dynamics in some segments of the population and identity markers (De Medeiros & Etter-Lewis, 2020). Scholar in life writing studies Gillian Whitlock builds further on this idea by arguing that life narratives help “personalize and humanize categories of people whose experiences are frequently unseen and unheard” (2007, p. 3). As Whitlock argues, there is an increasing interest in the lived experiences of people that illustrate a reflection of broader social and cultural changes.

Accordingly, life writing scholar Thomas G. Couser refers to these texts as “filial memoirs” (2009). The latter have become popular in South African literature over the past years. Examples include Carol Gibbs’ *All Things Bright and Broken* (2018), Julia Martin’s *The Blackridge House* (2019), Rofhiwa Maneta’s *A Man, A Fire, A Corpse* (2022), and Kabelo Chabalala’s *A Journey from Boyhood to Manhood* (2022). The thriving memoir industry in South Africa frames the genre’s vitality, as it “hold[s] a high historical and sociological significance” (Jacobs & Bank, 2019, p. 165). Its ability to capture the contemporary anxieties and concerns of individuals and communities gives it a wide of appeal.

Krog navigates the complexities of old age through a portrait of Serfontein’s day-to-day at the care home. Nursing reports frequently lack specific information regarding Serfontein’s mood. In fact, the reports evince the absence of a humanistic approach to dementia, underscoring that nurses limit themselves to medical tasks. Krog’s inclusion of the reports drops a hint at the idea that forms of more compassionate care and specific training on dementia care practices are needed. This is consistent with the recommendations of medical experts who provide guidance on the training requirements in care homes across the country (Brear et al., 2025; Jacobs et al., 2024). The argument is that “training nurses on dementia is not prioritized, with reluctance from both nursing schools and the regulating nursing council to include geriatrics in the curriculum” (Jacobs et al., 2024, p. 459). As a result, the nurses’ fixation on recording medical facts draws attention to Serfontein’s suppressed personhood and the pathologizing medical gaze (Burke, 2014). As an example, the word ‘patient’ – pt in the narrative – replaces Serfontein’s name throughout the story. As Krog notes:

12.10 Pt won’t eat. Said she felt ill. Blood pressure 140 over 70.

Dr suggested half a blood pressure pill otherwise she was too weak.

Matron gave Vit B12 injection.

15.00 Pt worked with typewriter. 2 x bowel movement. Dry and hard (2025, p. 24)

Nursing reports indicate that Serfontein is a body subjected to surveillance. Serfontein is devoid of any emotional support that extends beyond conventional gerontological practice. Serfontein’s conviction that “[she has] somebody with [her] all the time and [has] never been so alone” (Krog, 2025, p. 25) attests to it, despite the fact she is well-attended from a strictly clinical perspective. Serfontein is vocal about the loneliness that gradually overtakes her life in the care home, making explicit the inability of medical personnel to see her beyond their medical duties.

This element underscores how people with dementia “often possess a variety of competencies, including not only the capacity to value and to make decisions regarding their own lives” (Schou-Juul et al., 2025, p. 1). Serfontein is presented as an opinionated woman, who shows awareness of how institutionalization clearly marks the boundaries of health and illness (Grenier & Phillipson, 2023). Krog notes:

As you know, I fell. And to fall means you have to make a mental shift so that your body can reach your brain again. Don’t struggle up to go to the toilet, pee in your nappy. A mental shift. Let others wipe your arse. A tectonic mental shift (2025, p. 25).

In the care home, fears and longings arise from the silence of the society of well-being to which Serfontein belongs. Serfontein cannot avoid becoming embroiled in the attitudes that, in her view, blight South Africa. The care home, a built environment, compels Serfontein to reflect on the elements that make up the social fabric of South Africa, pointing out that “regard or disregard [is] probably the origin of endless destructiveness in South Africa” (2025, p. 69). The matriarch turns to language, as a means of coping, to deal with her turmoil and confusion.

Tiredness and boredom show that Serfontein’s notion of the self remains intact. This strategy is frequently her preferred option to express dissatisfaction. Relatedly, such a thing also showcases her vulnerability and kindness when confronted with challenges of later life in the care home. The sensation of tiredness and boredom acts as a stimulant that enhances Serfontein’s writing. She refuses to be washed and looked in every detail by her black carer with stoicism. This attitude highlights Serfontein’s necessity to be seen always in command even in the context of a care home. By giving prominence to Serfontein, Krog examines both the fluid and flexible nature of health identity construction. She accomplishes this in a familiar format, writing, which enhances this feature of her personality in the narrative. Thus, writing is a form of resistance on Serfontein’s side. She asserts her agency in spite of her diagnosis:

Pills: I have to drink heaps without knowing what they are called. So – it remains difficult to always remember how much I have to be THANKFUL for and how tired everyone is of my COMPLAINING and THANKLESSNESS, so don’t take this letter seriously. Point is, almost every one of my peers is dead. But as you correctly said last time: I mustn’t pretend that I once had hundreds of friends – yes, I always had to be enough for myself (Krog, 2025, p. 27, emphasis in the original).

These instances of lucidity throughout the narrative, whether consistently or in moments, demonstrate an articulated reasoning, at times. Through her writing, Serfontein expresses her dissatisfaction with her living situation in the care home. Once again, writing keeps Serfontein grounded and secure, enabling her to elaborate on both the realities and consequences of aging. In fact, it is through writing that Serfontein feels empowered and in command of the family, albeit away from the farm. The latter is a stronghold of Afrikaner culture. She clings on to the memories of the farm to cope with the routines of the care home. She urges Krog to do likewise in order to air her grievances. As Serfontein observes: “[i]f you feel I distort things, nothing prevents you from writing your own story correcting it” (Krog, 2025, p. 118). Serfontein tends to equate life at the care home with the idea that modern isolation as progress is a form of punishment. This phenomenon is further accentuated in later life, when old people are confined to the walls of the care home; thereby removing them from their sentimental and interpersonal possessions.

This aspect seems to trigger an inner sense of threat and insecurity in Serfontein. She feels the imposition of care home rules as a form of gradual dispossession. Serfontein is thus both an insider and an outsider to the group of residents in the care home. But remembering Elandspruit keeps her grounded:

As long as Elandspruit is not dispossessed, I can still get water from there, stretch my shortened sinews on its yard and purify my eyes on the familiar skyline (2025, p. 25).

Very often, (fear of) dispossession is conducive to depriving people with dementia from voicing their concerns (Baldwin, 2005, 2006), with caregivers nearly colonizing their minds. Yet, Serfontein maintains a wavy, sometimes bossy but elegant character. Collapse is her strategy for resistance, and only companionship helps her navigate change:

But suddenly I would give anything in the world to be there with the three of you. It is not that I wish you were here, it is that I want to be there, gathered in the tangible rustle of your wings. And this, for me, is the essence of ageing: not your used-out joints, but that you lose interest, lose the ability to create a life-pulse around you. That you simply want to cosset yourself in the energy radiating from being with others, essentially those of your own flesh and blood (2025, p. 120).

Central to this feeling is the dehumanization of aging, which takes the form of isolation and alienation in almost all its forms. Serfontein’s dementia-induced forgetting functions as a motif that mirrors her inability to be part of the new social fabric in South Africa. Serfontein embodies the intersection of South Africa’s haunting past, which is a remarkable feature in her characterization, and forgetting. These elements are sheltered by her retreat into older memories. This component further exacerbates her intense fears, occasional paranoia, and desire to inhabit a former era. This is best illustrated in her resistance to abandon the farm. This concern allows Krog for the exploration of questions of guilt and memory. The narrative thus moves away from her comfort zone territory into what becomes, as the narrative unfolds, a recurring preoccupation for Krog. This latter element reassures Serfontein of Krog’s affection. This is the case when Serfontein, in a fit of irritation typical of dementia in early stages, tells other residents and their visitors that nobody cares about her. As Krog recalls in a conversation with her sister Helena:

“That’s not a point at all! Ma’s head has been dangerously racketing for a while now. The beetles are loose there…She tells people she never sees her children and *you* live overseas. Such nonsense! One of us visits her almost every day, we make sure that she has a family dinner with one of us each weekend…When she talks such low snake shit, I can climb the walls” (2025, p. 174 emphasis in the original).

Serfontein and her daughters experience the reverse of roles. The more Serfontein deteriorates, the more Krog feels her presence is insufficient and Helena useless. As will be explored in the coming section, Krog faces the challenge of relocating herself on a map of symbolic and identity-based values.

Krog depicts dementia as a disruptive force that requires increasing professional care and attention with each passing day. This is evident when the sundown syndrome appears. Krog reports how “[Serfontein] is scared when the nurses prepare her for the night [and that it] must be soul-destroying” (2025, p. 179). Such a thing leads to a growing feeling of helplessness. This is further accentuated when Serfontein’s episodes of confusion become the norm rather than the exception. Serfontein’s cognitive decline ignites feelings of guilt and disappointment. Krog is not her family caretaker, and this infuriates Serfontein. The public exposure of Serfontein’s story lays bare her frailty. This element carries added emotional and ethical implications. To this end, Krog explores the whimsical nature of memory within the context of the care home. This elucidates the complexities surrounding their narrative identities as perceived in the microcosm of the care home.

## Memories in and from the Microcosm of the Care Home

The psychologist and essayist Carl Gustav Jung perceives the relationship between mother and daughter as cyclical and interconnected. In his influential work, *Aion: Researches into the phenomenology of the self* (1959)*,* the critic argues that a generational connection, as is the mother-daughter relationship, plays a key role in the perception of the sense of self, blending the past, the present and the future. Both this connection and emotional bondage, often seen as defining elements of the writings by women (Chaudhary & Dutt, 2025; Hanscombe, 2018; Jeremiah & Jordan, 2025; Oliveira et al., 2024; O'Neill & Schrage-Fruh, 2018; Zygadlo, 2012), acquire a complex dimension when the realities of aging and increasing levels of carework become the norm. As aging studies scholar Ulla Kriebernegg observes, there is a boom in the literary development of the so-called ‘care home narrative’ (2015, p. 159). *Blood’s Inner Rhyme* may well fall into this category, amongst others. It is in the care home where parts of the narrative play themselves out. In this sub-genre, family members trace the evolution of their relationship over time, examining the impact of mutual influence in their perception of identity. Krog writes about this bond, expressing Serfontein’s views, as follows:

One’s mother is the person one abandons many times over, but she: never. Forget the saying “we marry our mothers”. Perhaps a daughter shouldn’t know what she is internalizing of the mother…perhaps the most successful relationship is a non-relationship, a break out of each other’s grasps (2025, p. 39)

The care home, as the non-space, promotes a non-relationship or a replacement of it. Krog’s expresses the influence that they have on each other. This component anticipates the strained yet honest communication between Krog and Serfontein. Both master these instances that are rife with delicate balancing acts. As is sometimes the case, the specific conflicts presented in the narrative stem from different life choices and priorities in life, namely Krog’s balk at the idea of living in the farm, or else arguments over Serfontein’s level of independence. The latter is a reminder of how much care is required in Krog’s, Pieter’s, and Helena’s eyes. Serfontein tends to wipe off those remarks in her bid to preserve her authority and power in old age.

Serfontein’s authority showcases how Krog’s latest work does not follow the pattern of some other similar works in which the narrative predominantly “gives voice to the perspective of the daughter who speaks *for* her mother rather than engaging *with* her” (Tynan, 2025, p. 137, emphasis in the original). On the contrary, Serfontein remains mistress of the family until the very end:

A frantic, aggressive Afrikaner culture of survival was driven into us, physical survival, wrestled out with all available means through volks-pride. Sad ballads of starving mothers and child-victimhood were replaced with upbeat marching songs about spring and life in the open air (2025, p. 13).

Yet, these ideas are criticized and contested:

My and my mother’s politics had never gone together. Years ago, when I was still a young, fiery housewife here in Kroonstad, she used the k-word, which in those days was commonly used.

“I don’t want to hear that word in my house, ever,” I said to her.

She lost her temper. “Well, if I am not welcome in your house when I speak the way I speak, we essentially have nothing more to say to each other,” she shot back and stormed out.

Very early next morning […] [s]he swallowed. “I spent a long time thinking. You are right. This is your house, and you decide how you want in here and how people must behave. I will respect that. But then I expect that you also accept my choices in my house” (2025, pp. 45–46).

The narrative explores a number of tensions. These mostly stem from racial issues. Krog defies Serfontein’s influence, a woman whose political leanings tend to appreciate apartheid’s ‘enforced peace.’ Give-and-take plays a big part in their relationship, particularly when Krog visits her. The care home offers opportunities to overcome obstacles and the challenges that lie ahead. They discuss the (sub)conscious elements of their relationship, which are often codified in cherished memories. Memory is essential to grasp a better understanding of the position they occupy in life.

Krog and Serfontein pinpoint memories that encapsulate the nature of their bond. As dementia critical studies scholar Hedley Twidle points, “[s]pending time with someone with dementia dissolves the carer’s sense of identity” (2024, p. 57). In the portrayal of this relationship, Krog and Serfontein rely on memory to find shelter in each other. The ongoing negotiation of roles shapes their narrative identities. The latter are laced with instances of tension because, as Krog points, “Ma is the Empress of Insult” (2025, p. 177). This constant negotiation implies the exploration of their identities. These acquire greater significance in the context of disease, making room for unspoken thoughts and considerations. The care home environment fosters spaces that enhance interpersonal connections. As a result, instances of attentiveness and responsiveness emerge in the story. One such instance materializes in the representation of solitude. This aspect operates as a unifying element between them. For different reasons, they experience loss of interest and sometimes they sound aimless or without any further motivation. They discover refuge and tranquility in solitude. As is noted:

Both your letters arrived safely. What am I doing in Antwerp? I am what they call a writer-in-residence. There are many things here that remind me of you. To live alone for so long is more difficult than I thought. Initially I couldn’t understand the struggling […] I like being alone; like you. I was never surrounded by a group of friends. I am not a fearful person, or a chatterbox, so I thought being freed from my daily grind would be fantastic. Main issue is that I am no longer up to maintaining the antjie krog that I (or is it you?) brought into being. I am tired, especially tired of her. So, here I am, rid of her, anonymous. Why do I feel so unstable? (2025, p. 42).

The need for closeness and the drive for autonomy take over Krog’s heart and mind. Krog inhabits the realm of longingness, gaining greater awareness of her own sense of self. Navigating that path teaches Krog to prevent enmeshment, as the boundaries between their identities are anything but blurred. It encourages a different form of connection, a healthy solitude. This is essential for the formation of an identity that offers a safe foundation for the examination of their bond. Navigating solitude in this relationship is also crucial for Serfontein:

The cataract in my right eye is going to be done here locally […] Can’t imagine anything more useless because my left eye is still fine, but apparently reading is the only thing that helps against Alzheimer’s […] Have always believed that reading gives you access to a different, larger world. But these days that world is so overwhelming: in my kitchen, on TV, radio, newspaper, my stoep and view, my thoughts, my language, my tired butcher’s heart, that you sit at odds with this other world (2025, pp. 27–28).

Serfontein is afforded the opportunity to engage in self-reflection in the silence of the care home. The deterioration of her health, combined with the gradual loss of social networks as Serfontein grows older, compels her to confront the intricacies of old age in the care home (Ireland & Proulx, 2025).

Aging exposes the frailty of Serfontein. Bodily frailty urges Krog to engage in a process of profound reflection. This process reveals the challenges posed by stored-up emotions. Many of these derive from the sinking image of the South Africa of her mind, while some others stem from the country’s lack of specific course, which parallels Serfontein’s life in the care home. These examples constitute long-running disputes Serfontein and the rest of the world, and Krog delves into her mother’s disenchantment. Both the mother and daughter derive pleasure from tying up loose ends in their bid to achieve a sense of closure. This is compelling when it has the potential to bring them both peace and highlight what unites them. As Serfontein points out, “when I saw so many Dot Serfonteinisms in *your* work and some of our private family phantom(b)s, I thought, thus we constitute each other” (2025, p. 37, emphasis in the original).

Krog also embarks on a path of healing past wounds by “storying” Serfontein’s lived experiences. This aspect underscores the potential of narratives of aging and illness in and from the care home for transformative change. In this sense, *Blood’s Inner Rhyme* explores the fabric of life, namely the interconnectedness of individuals through a lens that is rife with introspection. It also revolves around the importance of becoming part of a network of human relations, and how the latter are interwoven to create unique and singular lived experiences.

For the purpose of researching into the significance of old age, authors often take readers into the heart of deep illness. This happens in Annie Ernaux’s *I Remain in Darkness* (1999) or Christine Bryden’s *Dancing with Dementia* (2005) and *Will I Still Be Me* (2018). In *Blood’s Inner Rhyme*, Krog explores the bond between a mother and a daughter through dialogical thinking with Serfontein. Their conversations evince attachment to land, foregrounding the symbolic value of the farm in Afrikaner culture. This reflects valuable insights into the construction of Serfontein’s and Krog’s narrative identity, with the focus on gaining mutual understanding.

Their ideas are mutually exclusive, and as Krog points out, “[t]he Krog family is not a democracy” (2025, p. 179). Serfontein acts on principle, rules with an iron fist and, sometimes, even with an acid tongue and mean disposition. It is popularly believed that friction brings affection. In Krog’s case, the sparks are also born from that friction, revealing geragogy is needed to tackle some topics. They grapple with one another to understand the rationale behind their thinking. Krog brings to Serfontein’s attention that black South Africans continue to face obstacles and effects of systemic violence. The consequences are made evident by the existence of social scars, which Serfontein tends to overlook, stating “the white men manipulate the narratives to make this thing you talk about today, reconciliation, possible” (2025, p. 90). Serfontein drops several broad hints at the idea that there is no hope in post-apartheid South Africa. As a result, Serfontein holds on to the symbols and values of the Afrikanerdom for the purpose of stability.

In the narrative, Serfontein operates as the custodian of family identity. This aspect is of utmost importance. It gives continuity to Serfontein’s life when the threat of identity fragmentation looms on the horizon due to the irreversible nature of dementia. In the context of early onset dementia, keeping the notion of life continuity represents a major challenge; simultaneously, living with dementia in the care home “provides new circumstances for meaning-making and structuring [their] self-stories” (Kłosińska & Leszko, 2023, p. 1). The exploration of such circumstances proves that their life stories are forged, contested and updated through dialogue, the interconnectedness of individuals, and the integration of those aspects that make us humans into one’s own narrative.

## Conclusions

This article contributes to the ongoing attempts to excavate the implications of aging in the context of gradual cognitive decline. I have also argued that Krog counterbalances stereotypes surrounding dementia that often exacerbate the idea that people living with dementia are stuck in dreamlike states. On the contrary, Serfontein’s voice in *Blood’s Inner Rhyme* showcases the interconnectedness of caring practices and human relations. Krog’s commitment reveals our obligations and responsibilities towards people who transition from old age to end-of-life (Sako & Falcus, 2022, p. 1). Her narrative constitutes a repository of public knowledge that is accessible to a wider audience on South African soil, allowing readers in this county to “narratively experience aging” (Kriebernegg, 2015, p. 179).

This manuscript offers a unique opportunity to test South Africa against the backdrop of aging from the perspective of medical humanities and narrative gerontology. By doing so, it offers future strength and direction to the emerging fields of aging studies and literary gerontology in the Southern African region. It also contributes to novel interventions that understand dementia beyond memory loss, as it is often interpreted in Euro-America and other contexts with more prosperous healthcare systems. For instance, to date, South Africa has not yet either drafted and implemented a national dementia/Alzheimer’s plan (Jacobs et al., 2024). The analysis of the growing number of dementia narratives in general, and dementia life narratives in particular, represents a great forum for opportunities at a national level to promote humanities-informed health campaigns around dementia/Alzheimer’s and strengthen the exchange between the South African state and the humanities.

Interventions may include using literary accounts of dementia to illustrate its intricacies at the local level. As mentioned earlier, the boom of life narratives in the post-apartheid era confirms that South Africans desire to learn more about the lived realities and experiences of the social groups with whom they barely interacted during apartheid (Jacobs & Bank, 2019). In South Africa, national initiatives can strengthen disparate communities with varying beliefs, providing a unifying national response to some of the shared challenges posed by dementia/Alzheimer’s: stigma, isolation, and the social death of the person with dementia. Such an initiative has the potential to bring together the personal and the national, showing how the act of remembering the lives of the people with dementia helps us face the unfinished business of building a democratic society. Although medical research remains without a shadow of a doubt critical, the current manuscript fosters an understanding of dementia as it takes shape in the South African literary landscape. It considers interdisciplinary approaches to comprehend dementia through the demographic singularities of the South Africa. This reveals the country is in dire need of developing its own dementia care models to distance itself from the atrocities of the past. This view, as was discussed, promotes a culture of shared experiences. Thus, the analysis of dementia life writing in South Africa and its contextual conditions develop within the fabric of the family, promoting dialogue and understanding within and outside institutionalized care. It renders visible the demographic shift in the country as well as the fact that aging is gaining momentum in literary discussions (Ortega Montero, 2025; Ortega Montero & Twidle, 2025; Ortega Montero, 2026; Twidle, 2024).

As previously stated, family reckonings result in inherited tensions that persist. In this context, mutual respect and conviviality between Serfontein and Krog shows the way to their counterparts. This serves as the driving force behind an emotional bond that is both reparative and restorative when old age-related conditions, such as dementia, compel individuals to clarify the values and beliefs that reformulate identity. Those instances of intimacy, confessions, solitude, and silence represent an opportunity to examine the emotional dimensions that we carry with ourselves in our lifetime, reflecting aspects of our lives that are deeply influenced by the impact of those around us.

*Blood’s Inner Rhyme* is a narrative that illustrates how dementia does not solely affect Dot Serfontein, but also those around her. This element has shown that it also dictates Krog’s sense of self. This powerful autobiographical work offers valuable insights into the resilience of being human in the context of illness, navigating the many challenges associated with end-of-life. As was shown earlier, the exploration of the interface between aging and family identity resulted in personal revelations that exemplify the qualities of introspection in overcoming the anxieties and tensions associated with age-related conditions. In this sense, autobiographical writing helps Krog gain insights into the power of reminiscence and life review.

## Data Availability

No datasets were generated or analysed during the current study.
